# P125: Promoting hand hygiene in intensive care: a permanent challenge

**DOI:** 10.1186/2047-2994-2-S1-P125

**Published:** 2013-06-20

**Authors:** D Joubert, C Landelle, E Genevois, J Pugin, S Harbarth, L Brochard, D Pittet

**Affiliations:** 1 Infection Control Program, University of Geneva Hospital, Geneva, Switzerland; 2 Intensive Care Unit, University of Geneva Hospital, Geneva, Switzerland

## Introduction

Healthcare workers (HCWs) compliance with hand hygiene (HH) remains a permanent challenge in ICUs and requires repeated adaptation of practices.

## Objectives

To monitor compliance with HH and appropriate use of gloves to help improving daily infection control practices.

## Methods

We monitored HH according to the WHO “My Five Moments for Hand Hygiene” concept and reviewed the appropriate use of gloves in a 36-bed mixed adult ICU admitting 2500 patients per year for an average length of stay of 3 days. The attack rate of methicillin-resistant *Staphylococcus aureus*(MRSA) cross-transmission was monitored based on active surveillance screening. In 2012, 9 % of admitted patients in the ICU carried MRSA. The intervention to improve HH practices included: simplifying the WHO “patient zone” concept in harmony with all wards at HUG; benchmarking of HH compliance with hospital-wide rates; promoting adherence with HH, with emphasis on improving adherence with the indication "before aseptic care"; stopping the routine use of gloves for "contact isolation" (except for *C. difficile*). Strategy implementation also included: additional installation and improved localization of alcohol-based handrub dispensers; teaching of glove use for paramedical staff; simulator training; and targeted training of new staff and clinical leaders.

## Results

A total of 1680 opportunities for HH were observed in 2011/2012. Overall compliance with HH improved from 51% in 2011 to 60% in 2012, and compliance with the WHO “before aseptic task" indication improved from 39% to 49%, respectively. Improvement was significant for all major HCW categories: nurses (from 59% to 65%), doctors (from 45% to 59%) and nursing assistants (from 41% to 66%). The MRSA attack rate decreased in parallel from 4.9 ICU-acquired cases/1000 patient-days in 2011 to 2.4 in 2012.

## Conclusion

The significant decrease of MRSA transmission associated with improved adherence to HH is encouraging. The 2013 target is to achieve an overall compliance with HH of 70% in ICU. Efforts will focus on a multimodal innovative approach.

## Disclosure of interest

None declared

